# Heterogeneity in Vulnerability and Resilience Among Centenarians

**DOI:** 10.1093/geroni/igab046.415

**Published:** 2021-12-17

**Authors:** Daniela Jopp, Charikleia Lampraki, Dario Spini

## Abstract

Given their exceptional longevity, centenarians have long been considered as examples of successful aging. Yet, with increases in empirical studies, findings suggest that they may show vulnerability and resilience at the same time. This symposium offers a more in-depth perspective on both constructs in centenarians. Zaccaria and colleagues investigated the link between social isolation and loneliness within the Fordham Centenarian Study. Results indicate the existence of four subgroups combining expressions of isolation and loneliness, suggesting different vulnerability patterns in centenarians. Uittenhove and colleagues analyzed patterns of coping strategies in the Second Heidelberg Centenarian Study. Cluster analysis identified two coping profiles, one characterized by a wide coping repertoire including problem-directed and internal strategies, while the other showed low problem-solving. Lampraki and Jopp examined the effects of (lacking) resources and psychological strengths (optimism) on depressive symptoms in the Fordham Centenarian Study. Findings suggest that the effect of resources is mediated by psychological strengths, demonstrating their beneficial value in very old age. Jopp and colleagues report findings from the ongoing SWISS100 Study. Based on telephone interviews conducted during the COVID-19 pandemic, they found that centenarians did not feel vulnerable. While half of the centenarians and their proxies reported no changes in everyday life, the other half experienced substantial challenges due to lack of activities and absence of social contacts due to governmental regulations. In sum, centenarians are vulnerable and resilient at the same time, highlighting the future research needs on its predictors, and the application of this knowledge within the context of crisis.

